# Data on the optimization of a GC–MS procedure for the determination of total plasma myo-inositol

**DOI:** 10.1016/j.dib.2016.07.024

**Published:** 2016-07-19

**Authors:** Jin Guo, Yingfei Shi, Chengbao Xu, Rugang Zhong, Feng Zhang, Bo Niu, Jianhua Wang, Ting Zhang

**Affiliations:** a Beijing Municipal Key Laboratory of Child Development and Nutriomics, Capital Institute of Pediatrics, Beijing 100020, China; b Beijing Key Laboratory of Environmental & Viral Oncology, College of Life Science and Bioengineering, Beijing University of Technology, Beijing 100124, China; c Chinese Academy of Inspection & Quarantine, Beijing 100023, China; d Department of Biochemistry and Molecular Biology, Shanxi Medical University, Taiyuan 030001, China

**Keywords:** Gas chromatography-mass spectrometry, Myo-inositol, Derivatives

## Abstract

Myo-inositol (MI) is one of the stereoisomers of hexahydroxycyclohexane, which plays an important role in intracellular signal pathway. Derivatization is an indispensable step in both external and internal standard method during the chromatography-mass spectrometer (GC–MS) detection, as MI can’t be ionized directly. It is valuable to optimize the derivative process and the detection volume for clinical detection. This article contains optimization data related to research publication “Quantification of plasma myo-inositol using gas chromatography–mass spectrometry” [1]. Here we introduce the data on the optimized derivatization volume, temperature, duration and the detection volume.

**Specifications Table**

**Table:** 

Subject area	*Chemistry*
More specific subject area	*Analytical Chemistry, Chromatography-Mass Spectrometer*
Type of data	*Table, graph*
How data was acquired	*GC–MS*
Data format	*Raw*
Experimental factors	*Extraction reagents were added to the plasma and evaporated to be dry before derivatization;* experimental factors include the derivatization volume, temperature, duration and the detection volume.
Experimental features	*7890A Gas Chromatography equipment a fused silica HP- 5 MS capillary column (Agilent Technologies, USA) was used for the GC separation.*
Data source location	*Beijing, China*
Data accessibility	*Data with this article*

**Value of the data**

• The data for the optimized derivatization volume, temperature, duration and the detection volume is presented;
• Periphery blood is enough for the detection of plasma MI;
• The optimized derivatization condition for MI analysis could be used not only for biological specimens, but also for food and others.

## 1 Data

**Fig. 1:**
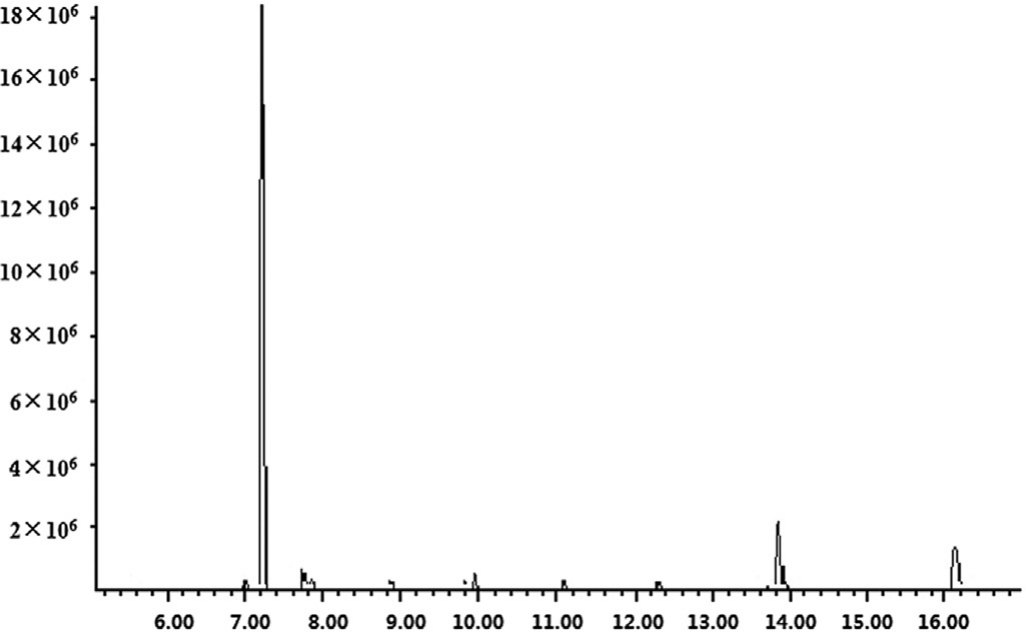
Total ion chromatogram (TIC) profiles of GC–MS results.

**Fig. 2:**
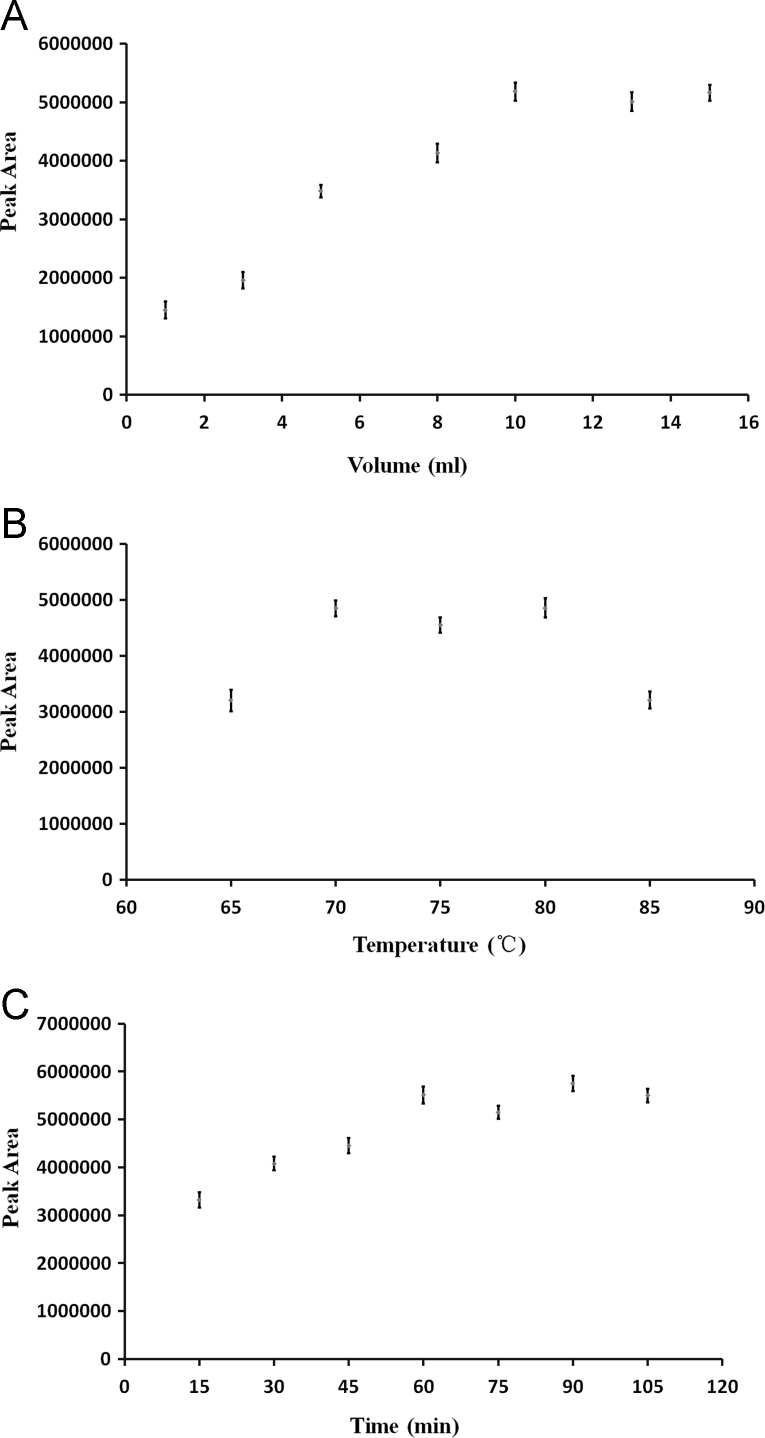
Optimal derivatization conditions for plasma MI detection. The derivatization reagent amount (A) derivative temperature (B) and derivative time (C) were optimized.

This data consist of the optimal derivatization condition of MI, including amount of derivatization reagent, derivative temperature and derivative time (Fig. 1, Fig. 2). Furthermore, the detection volume of the plasma was minimized (Table 1).

## 2 Experimental design, materials and methods

### 2.1 Gas chromatography instrument and reagents

The gas chromatography instrument and reagents were used as our previous study [1].

### 2.2 Optimal derivatization condition of MI

Derivatization is an important and vital step in pretreatment process in GC–MS analysis [2], [3]. Derivatization conditions were optimized by using a 5 mg/l MI working solution, which was placed into 30 μl human plasma. The various amount of derivatization reagent (1 ml, 3 ml, 5 ml, 8 ml, 10 ml, 13 ml and 15 ml), derivative temperature (65 °C, 70 °C, 75 °C, 80 °C and 85 °C) and derivative time (15 min, 30 min, 45 min, 60 min, 75 min, 90 min and 105 min) were investigated. The peak area of various derivatization conditions was analyzed and results were shown in Fig. 1. It was found that the derivatization yield increased as the increase of reaction volume less than 5 ml. 5 ml reagent was observed to be sufficient for derivatization. So did the situation when the derivative temperature above 70 °C and the derivative duration longer than 60 min (Fig. 2). Therefore, it was determined that the optimized derivatization conditions were using a 5 ml mixture of TMCS/HMDS/N, N - DMF at 70 °C for 60 min and shaking at 10 min intervals.

### 2.3 Optimal the volumes of plasma for detection

To minimize the detection volume of the plasma, various volumes of plasma samples (10 μl, 30 μl, 50 μl and 70 μl) were pretreated and evaluated by GC–MS method. The result showed that the plasma volume was proportional to the concentration of the plasma MI from 70 μl to 30 μl of the detection volume. Therefore, 30 μl was the minimal volume for detection (Table 1).

## Figures and Tables

**Table 1:** Optimize the plasma volume for MI detection.

Injection volume (μL)	Peak area	MI concentrion (μg/L)	Ratio (MI concentrion/Injection volume)
10	1142317	5	0.50
30	2205462	87	2.90
50	2556501	136	2.72
70	3211173	181	2.59
